# Supplementary material for: Long-read metagenomics using PromethION uncovers oral bacteriophages and their interaction with host bacteria
Source: Nat Commun. 2021 Jan 4;12:27. doi: 10.1038/s41467-020-20199-9 (PMC7782811; doi:10.1038/s41467-020-20199-9)
Supplement: Supplementary file 1 — Supplementary Information [file 41467_2020_20199_MOESM1_ESM.pdf]

Supplementary information for

Long-read metagenomics using PromethION uncovers novel oral bacteriophages, their abundance, and interaction with host bacteria

Koji Yahara[1*], Masato Suzuki[1], Aki Hirabayashi[1], Wataru Suda[2], Masahira Hattori[2], Yutaka Suzuki[3], Yusuke Okazaki[4]

[1] Antimicrobial Resistance Research Center, National Institute of Infectious Diseases, Tokyo, Japan; [2] Laboratory for Microbiome Science, RIKEN Center for Integrative Medical Sciences, Kanagawa, Japan; [3] Laboratory of Systems Genomics, Department of Computational Biology and Medical Sciences, Graduate School of Frontier Sciences, The University of Tokyo.; [4] Bioproduction Research Institute, National Institute of Advanced Industrial Science and Technology, Tsukuba, Japan

*Correspondence to: Koji Yahara, Antimicrobial Resistance Research Center, National Institute of Infectious Diseases, Aoba-cho 4-2-1, Higashimurayama-shi, Tokyo, 189-0002 Japan. Tel: +81-42-202-6080. Email: k-yahara@nih.go.jp

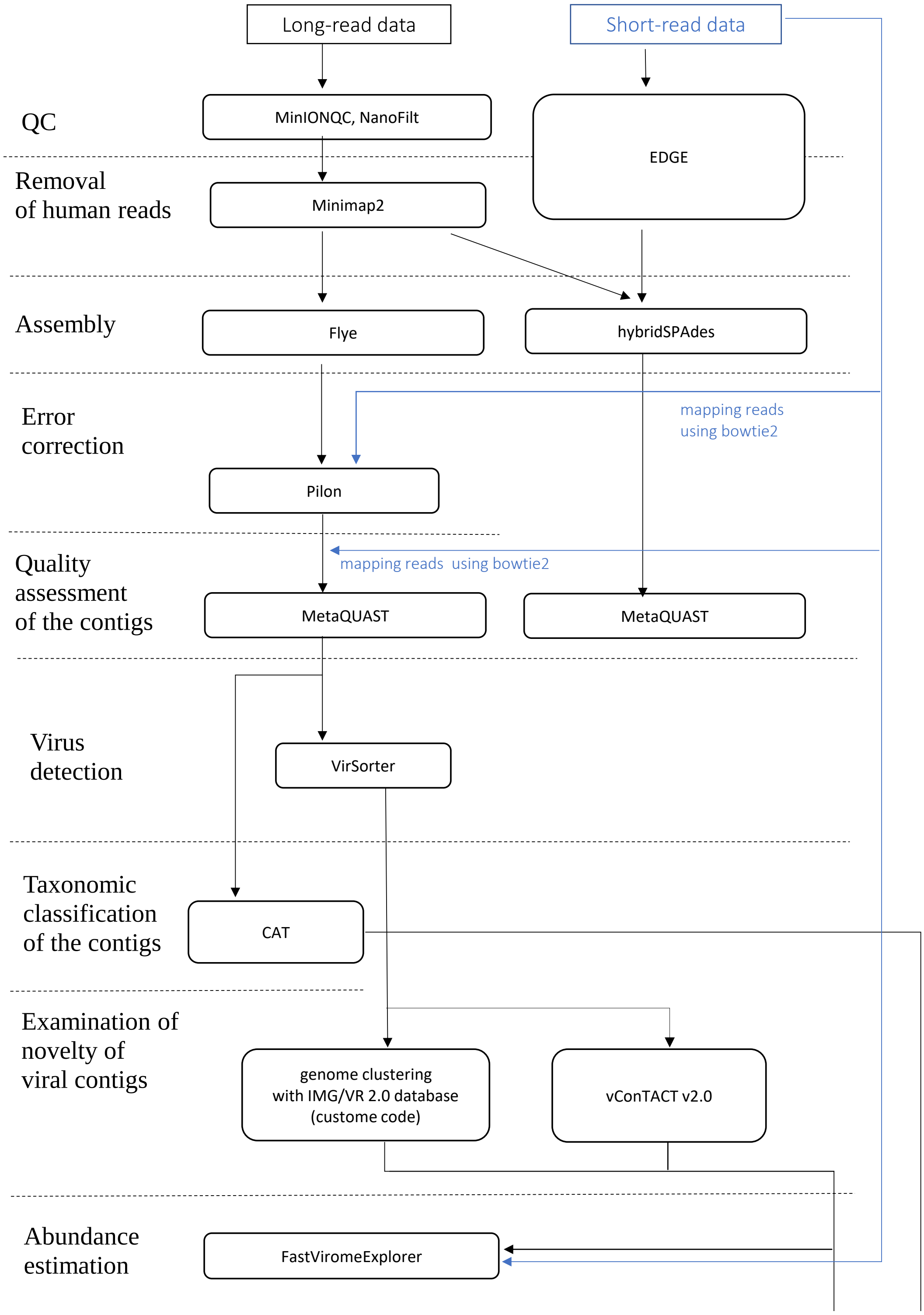

Long-read data
Short-read data
QC
MinIONQC, NanoFilt
EDGE
Removal
of human reads
Minimap2
Assembly
Flye
hybridSPAdes
Error
correction
mapping reads
using bowtie2
Pilon
Quality
assessment
of the contigs
mapping reads using bowtie2
MetaQUAST
MetaQUAST
Virus
detection
VirSorter
Taxonomic
classification
of the contigs
CAT
Examination of
novelty of
viral contigs
genome clustering
with IMG/VR 2.0 database
(custome code)
vConTACT v2.0
Abundance
estimation
FastViromeExplorer

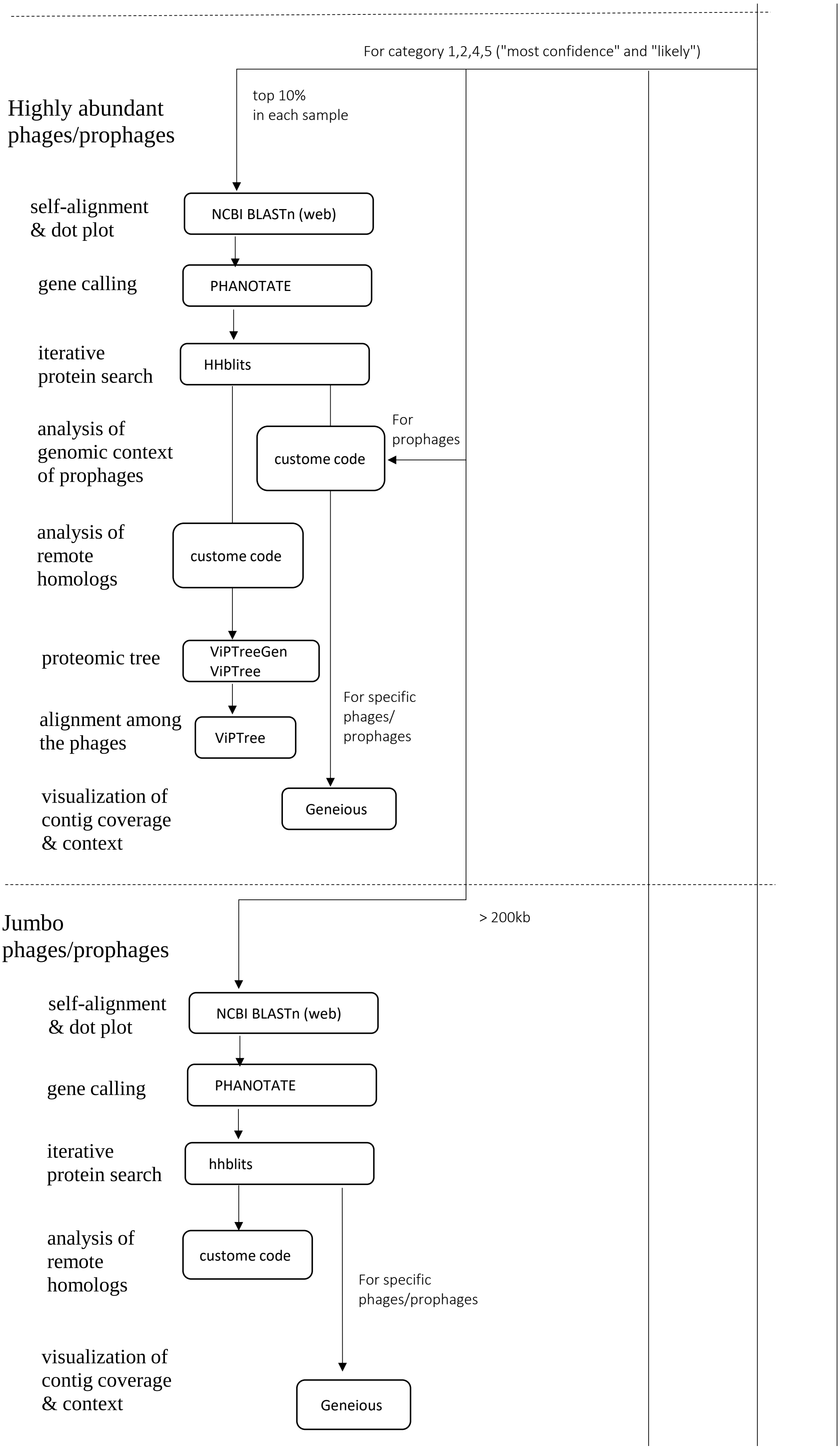
For category 1,2,4,5 ("most confidence" and "likely")
Highly abundant phages/prophages
top 10% in each sample
self-alignment & dot plot
NCBI BLASTn (web)
gene calling
PHANOTATE
iterative protein search
HHblits
analysis of genomic context of prophages
custome code
For prophages
analysis of remote homologs
custome code
proteomic tree
ViPTreeGen ViPTree
alignment among the phages
ViPTree
For specific phages/ prophages
visualization of contig coverage & context
Geneious
Jumbo phages/prophages
> 200kb
self-alignment & dot plot
NCBI BLASTn (web)
gene calling
PHANOTATE
iterative protein search
hhblits
analysis of remote homologs
custome code
For specific phages/prophages
visualization of contig coverage & context
Geneious

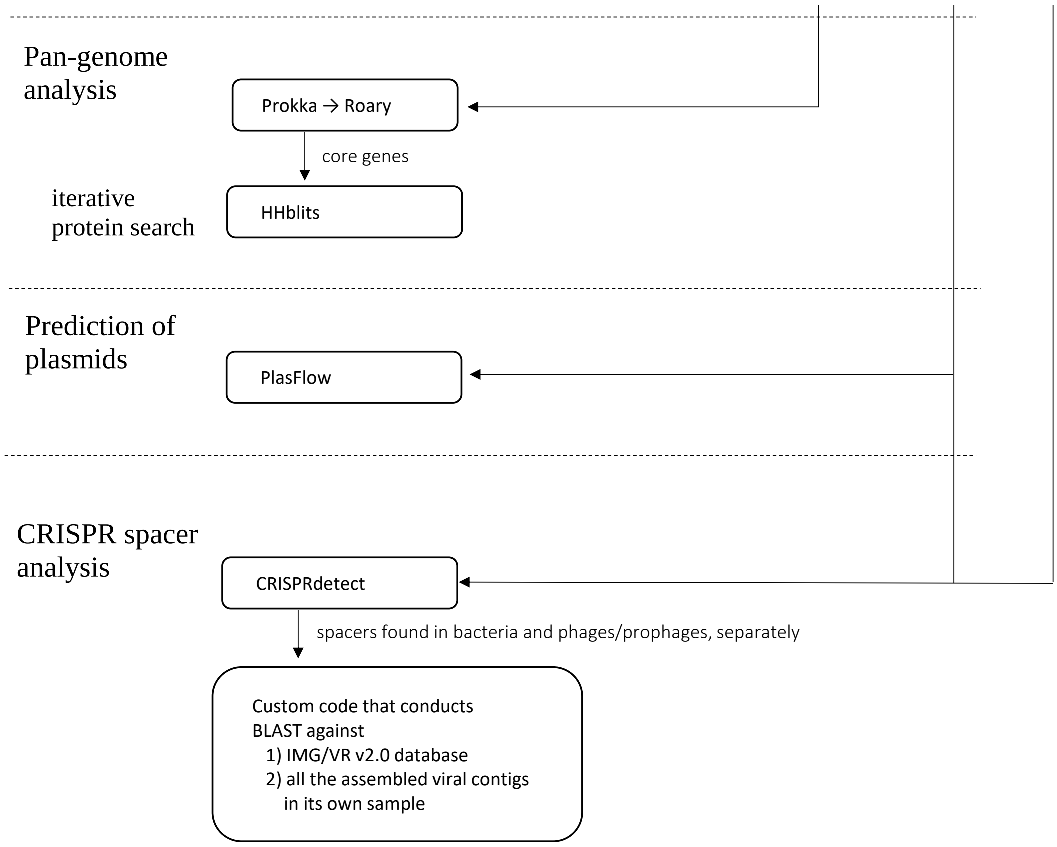

Supplementary Figure 1. Bioinformatic workflow.

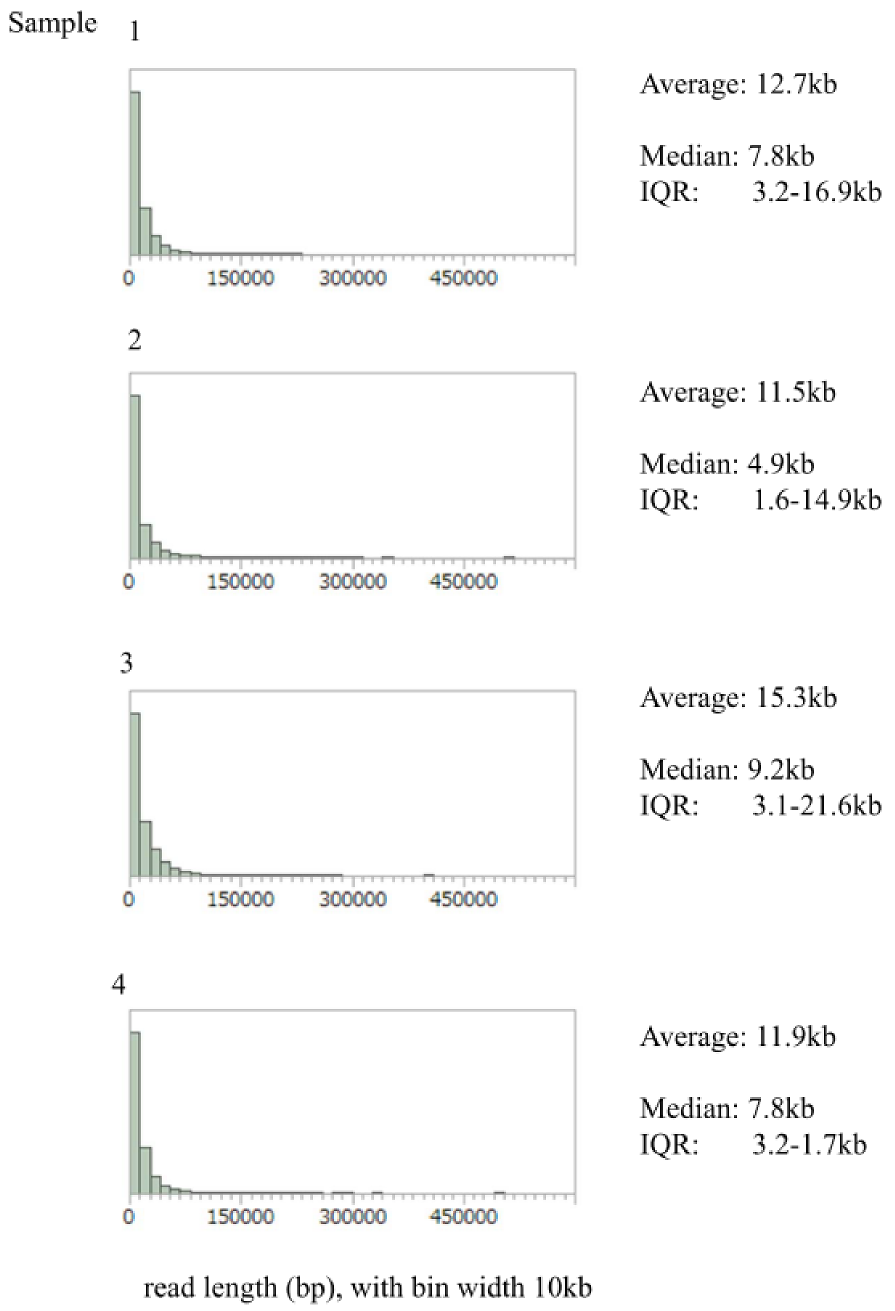

Supplementary Figure 2. Read length distribution. The histogram is shown for each sample separately, with bin width 10 kb.

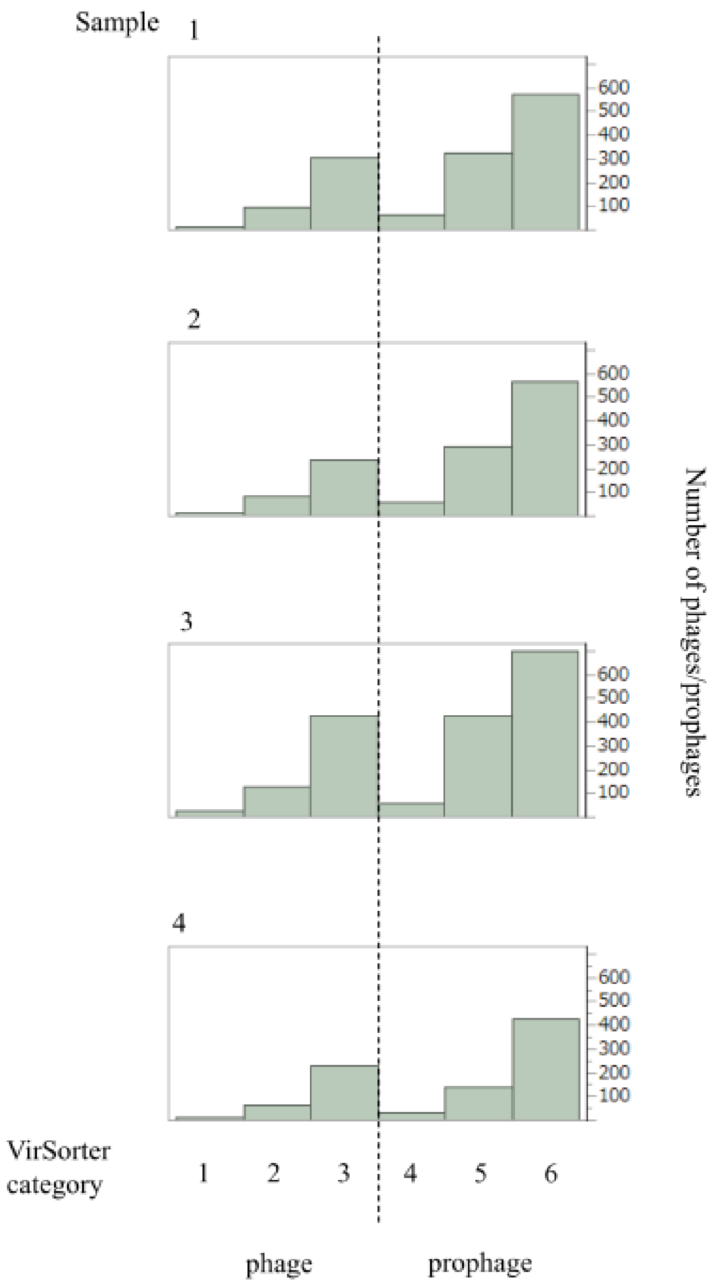

Supplementary Figure 3. The number of viral sequences identified in the contigs assembled from the long-reads of each sample according to the VirSorter categories.

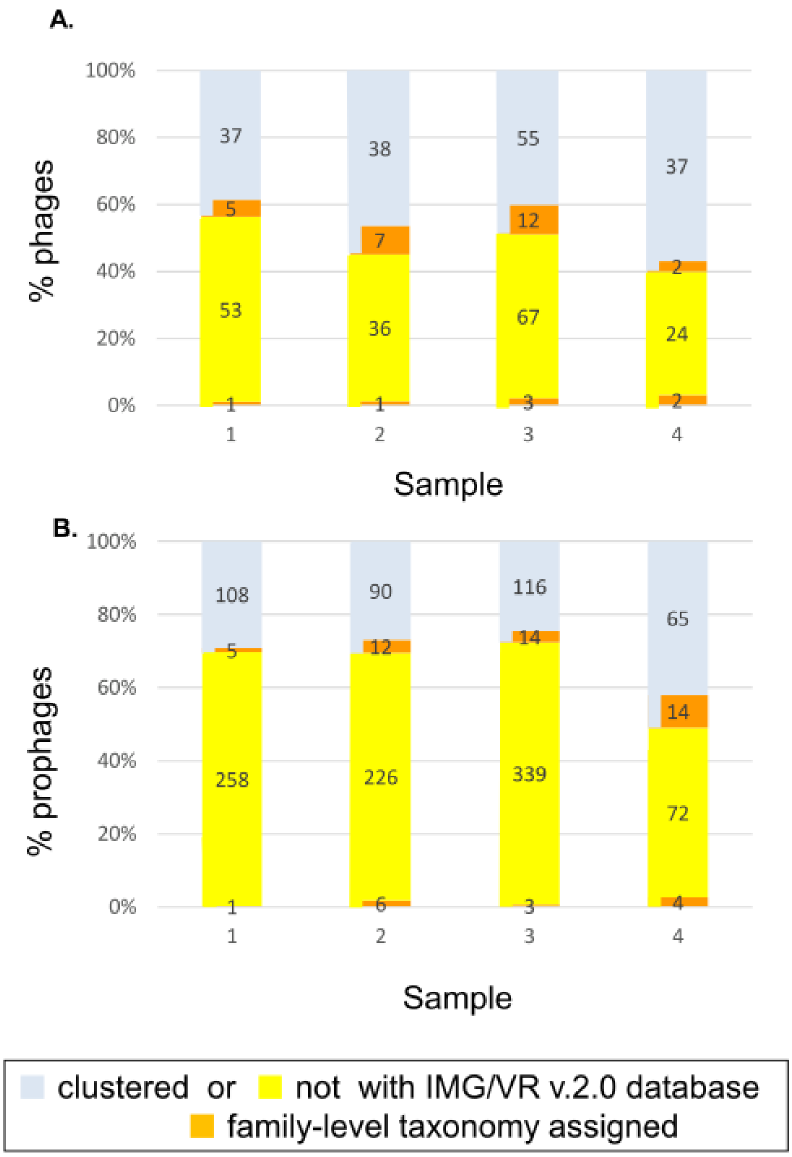

Supplementary Figure 4. The number and proportion of viral sequences of (A) phages and (B) prophages that were classified at the family-level. Orange: the taxonomic assignment was possible at the family-level. Yellow: not clustered with viral sequences in the IMG/VR v2.0 database, and regarded as the novel**.**

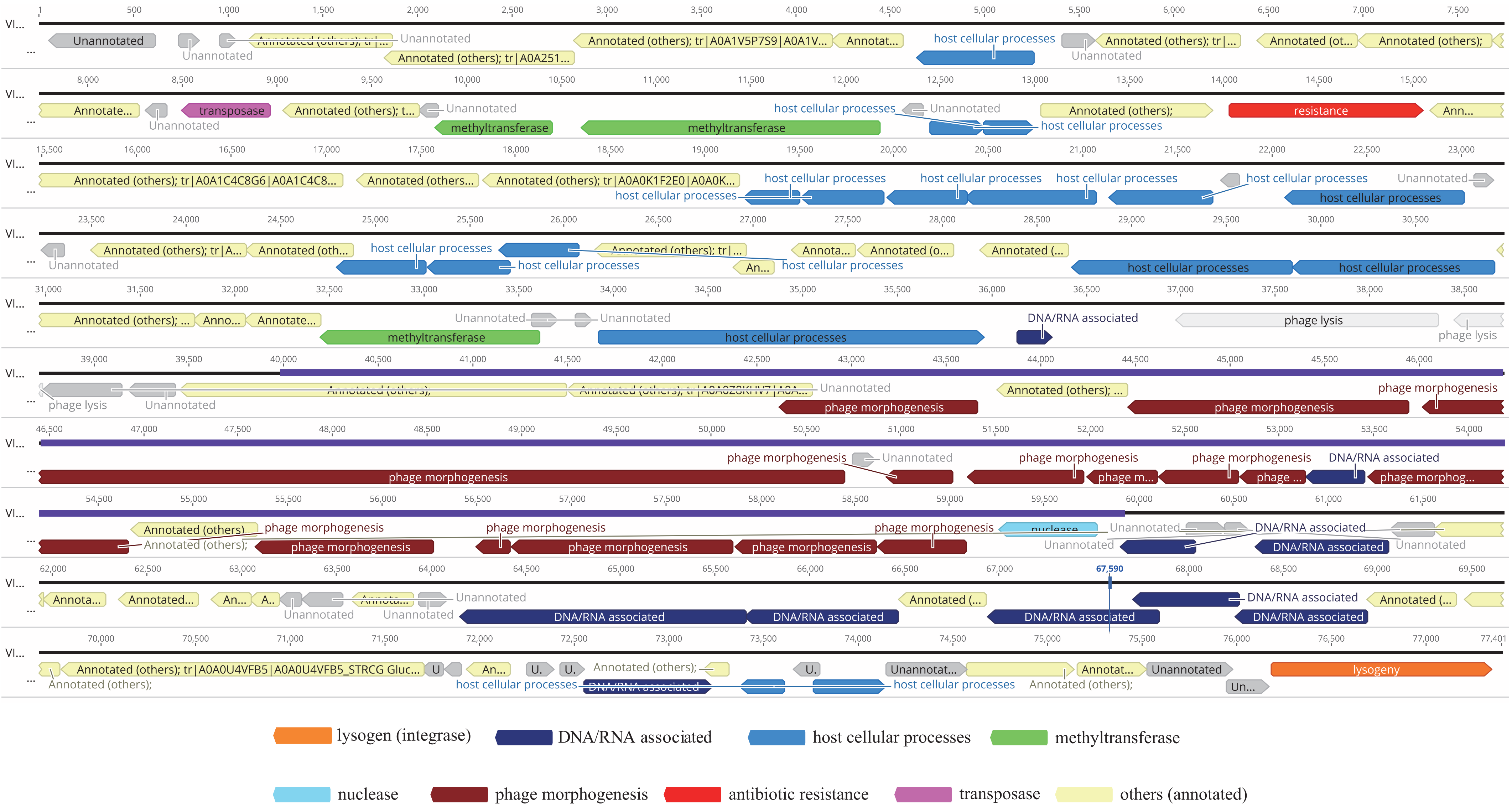

Supplementary Figure 5. An enlarged genome map of an oral prophage with the enhanced scaffolding and its host genomic context. Genes characterized by the HMM-based iterative protein searches are shown with colors according to their functional categories, for the prophage shown in Figure 2. The purple horizontal line corresponds to the region aligned with a virus registered in IMG/VR v 2.0 database in Figure 2.

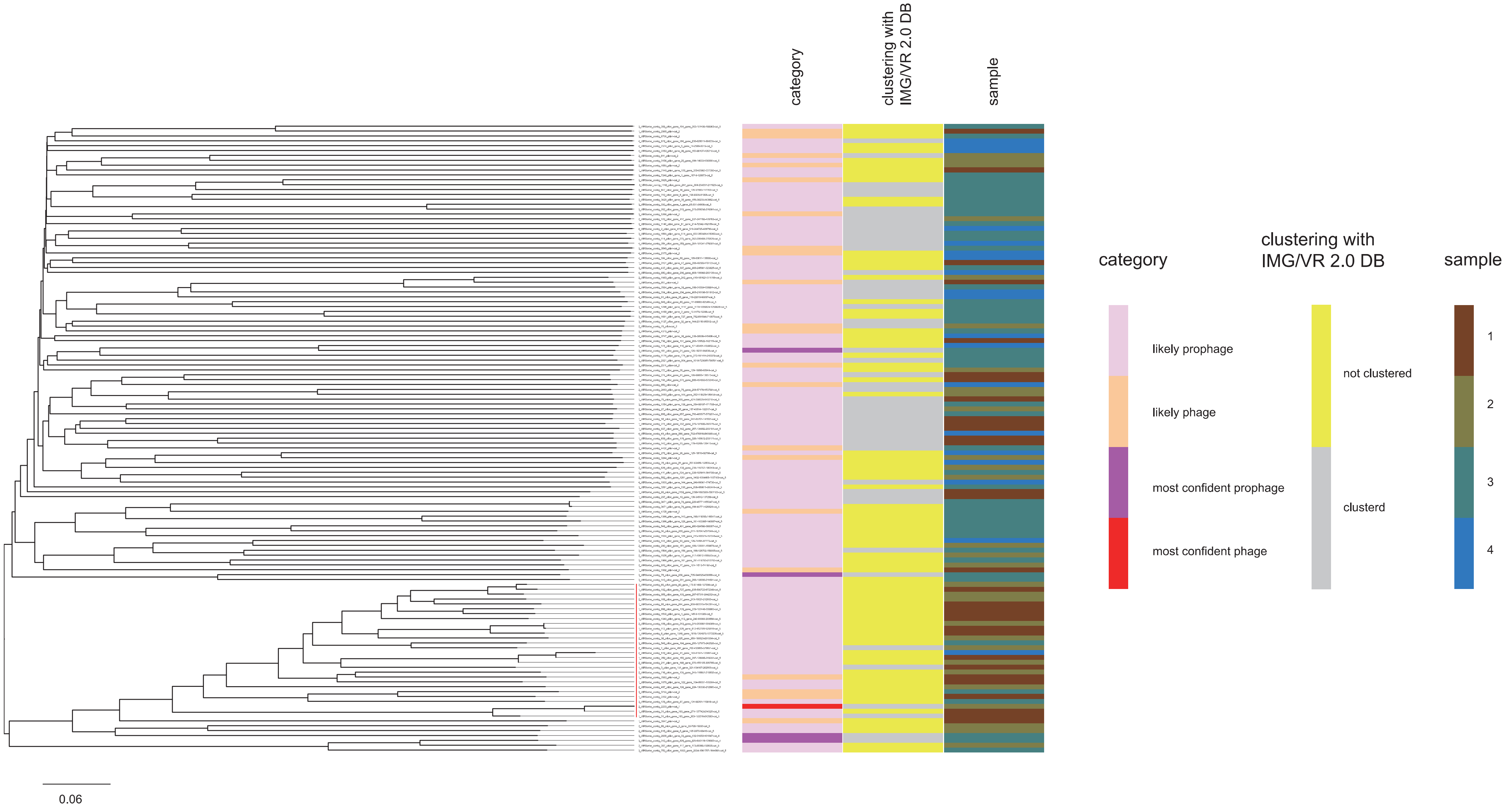

Supplementary Figure 6. A proteomic tree of the highly abundant (top 10%) phages/prophages . The color bars indicate viral categories classified by VirSorter, whether each phage/prophage clusters with viral sequences in the IMG/VR 2.0 database, and samples. The red vertical line indicates the cluster examined in the main text.

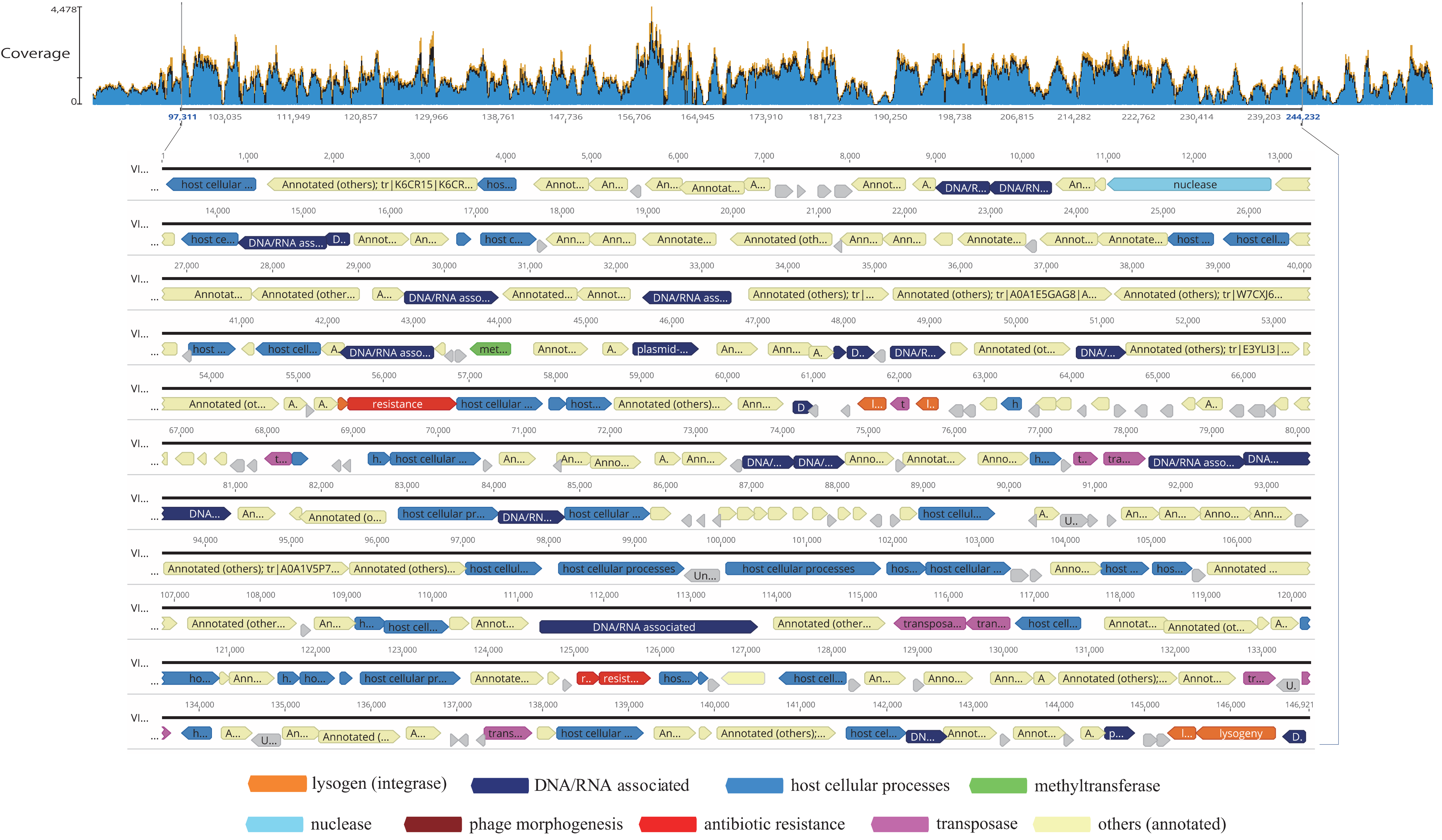

Supplementary Figure 7. An enlarged genome map of an oral *Siphoviridae* prophage. Top: coverage plot based on mapping short-reads against it. Bottom: Genes characterized by the HMM-based iterative protein searches are shown with colors according to their functional categories.

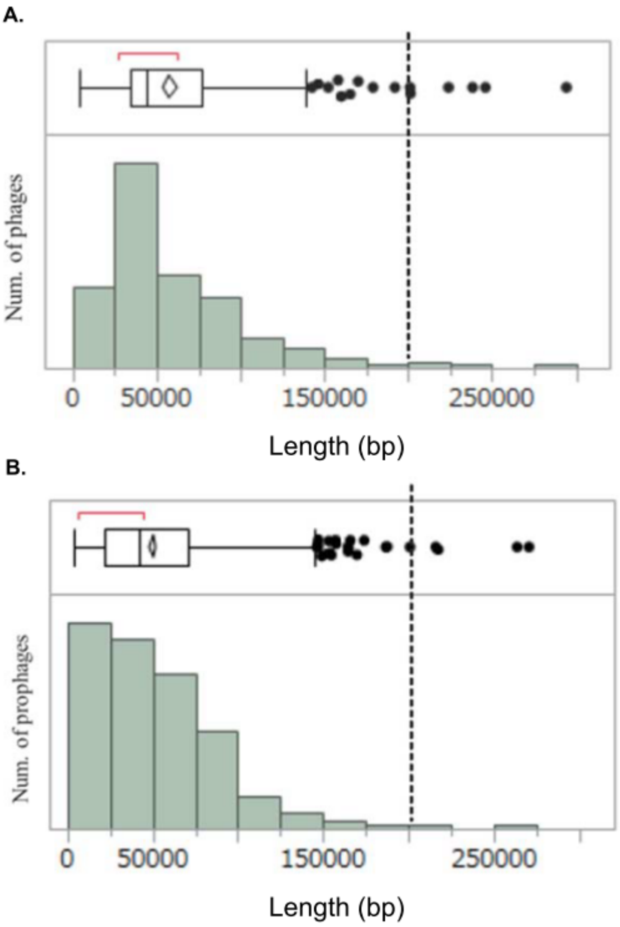

Supplementary Figure 8. Length distribution of viral sequences classified into (A) phages and (B) prophages. The dashed vertical lines indicate 200 kb as a standard cutoff to define jumbo phages/prophages. In the box plot, left and right of the box indicate 25th and 75th percentile, horizontal line indicates median, the middle of the diamond indicates average, the right outliers are above 75th percentile + 1.5 × interquartile range, and the red indicates the shortest range in which half of the data distributes. n=380 phages in (A) and n=1333 prophages in (B).

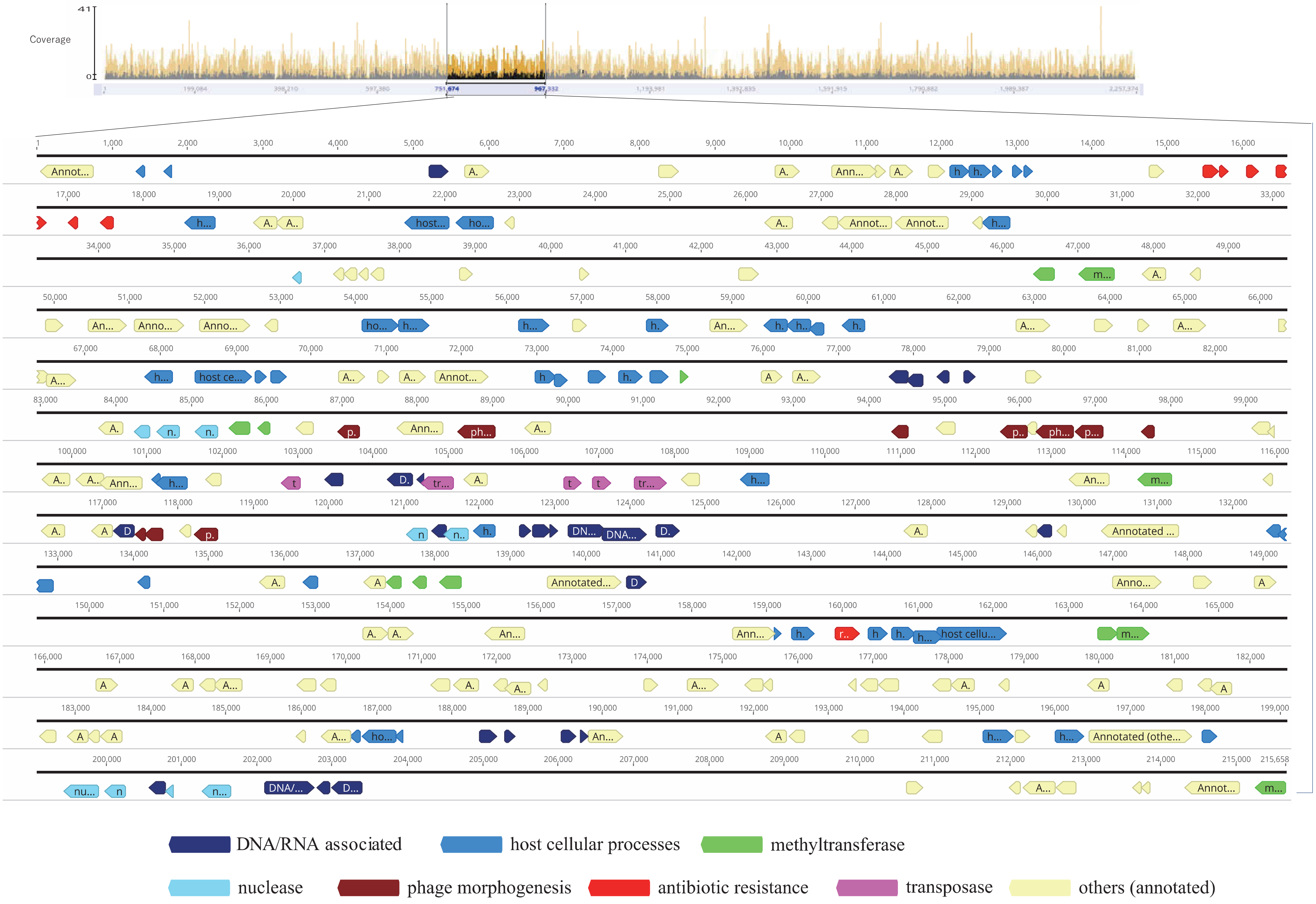

Supplementary Figure 9. An enlarged genome map of a jumbo prophage. This figure is similar to Supplementary Figures 5 and 7. The prophage region is indicated as a rectangle in the coverage plot spanning the entire contig.

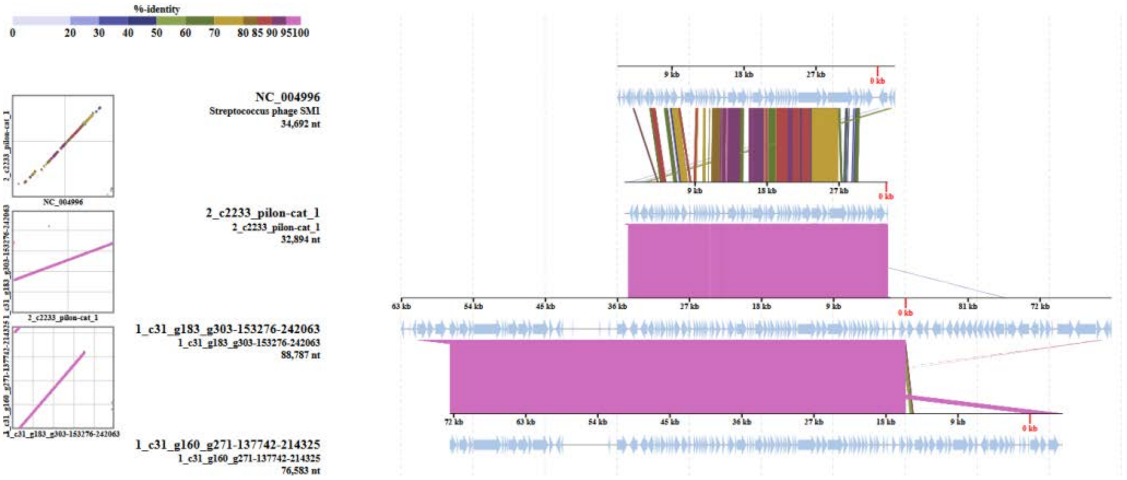

Supplementary Figure 10. Genome alignment of streptococcal phages. The top is a known phage SM1 (NC_004996). The 2nd is the "most confident" phage while the 3rd and 4th are the "likely" prophages identified in this study and included in the cluster of highly abundant phage/prophages The prefix numbers ("2_" and "1_") indicate sample numbers, and "c" and "g" represents "contig" and "gene," respectively.

Supplementary Tables

Supplementary Data 1-6 are available as spreadsheets.
