# Supplementary material for: Long-read metagenomics using PromethION uncovers oral bacteriophages and their interaction with host bacteria
Source: Nat Commun. 2021 Jan 4;12:27. doi: 10.1038/s41467-020-20199-9 (PMC7782811; doi:10.1038/s41467-020-20199-9)
Supplement: Supplementary file 3 — Description of Additional Supplementary Files [file 41467_2020_20199_MOESM3_ESM.docx]

File Name: Supplementary Data 1
Description: Spreadsheet showing concentration of the DNA extracted from the saliva samples

File Name: Supplementary Data 2
Description: Spreadsheet showing the amount of sequence data generated

File Name: Supplementary Data 3
Description: Spreadsheet showing assembly statistics calculated using MetaQuast

File Name: Supplementary Data 4
Description: Spreadsheet showing viral sequences identified by VIRSorter

File Name: Supplementary Data 5
Description: Spreadsheet showing *Streptococcus* phages/prophages group found across the 4 samples

File Name: Supplementary Data 6
Description: Spreadsheet showing the core genes and their annotation by HHblits
