# Supplementary material for: Long-read metagenomics using PromethION uncovers oral bacteriophages and their interaction with host bacteria
Source: Nat Commun. 2021 Jan 4;12:27. doi: 10.1038/s41467-020-20199-9 (PMC7782811; doi:10.1038/s41467-020-20199-9)
Supplement: Supplementary file 10 — Reporting Summary [file 41467_2020_20199_MOESM10_ESM.pdf]

nature research

Corresponding author(s): Koji Yahara

Last updated by author(s): 12 November, 2020

Reporting Summary

Nature Research wishes to improve the reproducibility of the work that we publish. This form provides structure for consistency and transparency in reporting. For further information on Nature Research policies, see our Editorial Policies and the Editorial Policy Checklist.

Statistics

For all statistical analyses, confirm that the following items are present in the figure legend, table legend, main text, or Methods section.

n/a	Confirmed	
☐	☒	The exact sample size (*n*) for each experimental group/condition, given as a discrete number and unit of measurement
☐	☒	A statement on whether measurements were taken from distinct samples or whether the same sample was measured repeatedly
☐	☒	The statistical test(s) used AND whether they are one- or two-sided<br>*Only common tests should be described solely by name; describe more complex techniques in the Methods section.*
☒	☐	A description of all covariates tested
☒	☐	A description of any assumptions or corrections, such as tests of normality and adjustment for multiple comparisons
☒	☐	A full description of the statistical parameters including central tendency (e.g. means) or other basic estimates (e.g. regression coefficient) AND variation (e.g. standard deviation) or associated estimates of uncertainty (e.g. confidence intervals)
☐	☒	For null hypothesis testing, the test statistic (e.g. *F*, *t*, *r*) with confidence intervals, effect sizes, degrees of freedom and *P* value noted<br>*Give P values as exact values whenever suitable.*
☒	☐	For Bayesian analysis, information on the choice of priors and Markov chain Monte Carlo settings
☒	☐	For hierarchical and complex designs, identification of the appropriate level for tests and full reporting of outcomes
☒	☐	Estimates of effect sizes (e.g. Cohen's *d*, Pearson's *r*), indicating how they were calculated

Our web collection on statistics for biologists contains articles on many of the points above.

Software and code

Policy information about availability of computer code

Data collection	no software was used
Data analysis	EDGE pipeline (v1.5); MinIONQC (v1.3.5); NanoFilt (v2.7.1); bowtie2 (v2.2.4); minimap2 (v2.15); Flye (v2.4.2); SPAdes (v3.13.0) ; pilon (v1.23); MetaQUAST (v5.0.2); VirSorter (v1.0.5 installed using conda on Mar 29, 2019); CAT (v4.5); mcl (v14-137); vConTACT v2.0; bbmap (v 38.68); kallisto (v 0.43.1); FastViromeExplorer (v1.2); PHANOTATE included in multiPhATE v.1.0; HHblits (v3.1.0); VIPTreeGen (v1.1.2); Prokka (v1.13); Roary (v1.006925); PlasFlow (v1.1); CRISPRdetect (v2.2); samtools (v1.9); Custom codes were used as explained at https://github.com/bioprojects/PromethION-oral-phageome-paper

For manuscripts utilizing custom algorithms or software that are central to the research but not yet described in published literature, software must be made available to editors and reviewers. We strongly encourage code deposition in a community repository (e.g. GitHub). See the Nature Research guidelines for submitting code & software for further information.

Data

Policy information about availability of data

All manuscripts must include a data availability statement. This statement should provide the following information, where applicable:

- Accession codes, unique identifiers, or web links for publicly available datasets
- A list of figures that have associated raw data
- A description of any restrictions on data availability

The data of PromethION and HiSeq after the quality control and removal of human reads were deposited at DDBJ (with JGA accession number JGAS00000000186) and is mirrored at NCBI under BioProject accession PRJDB9452 [https://www.ncbi.nlm.nih.gov/bioproject/?term= PRJDB9452]. The data of HiSeq of the additional experiment after the quality control and removal of human reads were deposited at DDBJ and is mirrored at NCBI under BioProject accession PRJDB10605 [https://www.ncbi.nlm.nih.gov/bioproject/?term=PRJDB10605]. All nucleotide sequence data provided under the Figshare link were deposited at DDBJ (with accession

numbers BNJS01000001-BNJS01004500, BNJT01000001-BNJT01003334, BNJU01000001-BNJU01006278, and BNJV01000001-BNJV01003374 for each sample, respectively). IMG/VR v2.0 [https://img.jgi.doe.gov/vr/] and UniProt [https://www.uniprot.org/] databases were used. Source data are provided with this paper.

Field-specific reporting

Please select the one below that is the best fit for your research. If you are not sure, read the appropriate sections before making your selection.

[x] Life sciences [ ] Behavioural & social sciences [ ] Ecological, evolutionary & environmental sciences

For a reference copy of the document with all sections, see nature.com/documents/nr-reporting-summary-flat.pdf

Life sciences study design

All studies must disclose on these points even when the disclosure is negative.

Sample size	The sample size was determined based on a limited budget for obtaining the >30Gb metagenomic data of both PromethION and HiSeq per sample. It does not enable to statistically test between-sample difference, but still enables to explore whether consistent patterns were found across the samples, and proportion of the core and singleton genes encoded in oral phages/prophages.
Data exclusions	Data of reads with low quality and mapped to the human genome were excluded from the analysis in the preprocessing step (Methods) using the criteria defined in the EDGE pipeline in advance.
Replication	We have replicated twice the data analyses using the custom codes according to procedures in https://github.com/bioprojects/PromethION-oral-phageome-paper.
Randomization	This is not relevant because there is no group allocation and between-group comparison in this study.
Blinding	This is not relevant because there is no group allocation and between-group comparison in this study.

Reporting for specific materials, systems and methods

We require information from authors about some types of materials, experimental systems and methods used in many studies. Here, indicate whether each material, system or method listed is relevant to your study. If you are not sure if a list item applies to your research, read the appropriate section before selecting a response.

Materials & experimental systems

n/a	Involved in the study	
[x]	[ ]	Antibodies
[x]	[ ]	Eukaryotic cell lines
[x]	[ ]	Palaeontology and archaeology
[x]	[ ]	Animals and other organisms
[ ]	[x]	Human research participants
[x]	[ ]	Clinical data
[x]	[ ]	Dual use research of concern

Methods

n/a	Involved in the study	
[x]	[ ]	ChIP-seq
[x]	[ ]	Flow cytometry
[x]	[ ]	MRI-based neuroimaging

Human research participants

Policy information about studies involving human research participants

Population characteristics	Healthy Japanese volunteers (2 men and 2 women aged 35 to 65 years old)
Recruitment	The participants were recruited among workers in National Institute of Infectious Diseases and their relatives by direct invitations from the corresponding author. Potential biases are 1) two participants (No.1 and No.2) were living together, and 2) three participants (No.1-3) were scientists. Therefore, oral phageome of participants No.1-2 or that of participants No.1-3 might be similar compared to the others, although we did not find such a result in this study.
Ethics oversight	IRB of National Institute of Infectious . Written informed consent was obtained as described in the Methods section.

Note that full information on the approval of the study protocol must also be provided in the manuscript.
